# Prevalence of Depression and Suicidality Among Children and Adolescents with Sickle Cell Anaemia in Eastern Uganda

**DOI:** 10.21203/rs.3.rs-9798756/v1

**Published:** 2026-06-23

**Authors:** Dedan Okello, Lisa Rynn, Milton W. Musaba, Sarah Kiguli, David Mukunya, Joseph Kirabira

**Affiliations:** Busitema University; SEED GLOBAL HEALTH UGANDA; Busitema University; Makerere University; Busitema University; Busitema University

**Keywords:** Depression, suicidality, Sickle cell anaemia, Children, Adolescents, Eastern Uganda, Mbale Regional Referral Hospital, Busitema University

## Abstract

**Background:**

Depression and suicidality are prevalent among individuals with chronic illnesses. Data on their prevalence in children and adolescents with sickle cell anaemia in Uganda is limited. This study aimed to determine the prevalence and associated factors of depression and suicidality among children with SCA in Eastern Uganda.

**Methods:**

We consecutively enrolled children with SCA between 9 and 17 years, at Mbale Regional Referral Hospital between 01/02/2024 and 05/06/2024. Depression and suicidality were measured using the MINI KID. Data were collected using an interviewer administered questionnaire and entered into KoboToolbox for electronic data management. Analysis was performed using Stata 17. Bivariate and multivariable logistic regression analyses were done to examine factors associated with depression and suicidality.Statistical significance was assessed at the 0.05.

**Results:**

421 participants were enrolled, the prevalence of depression was 31.8%, while suicidality was 19.0%. The Factors associated with depression, were secondary level of education (AOR: 3.5, 95% CI: 1.965–12.756; p = 0.049), Children with formally employed parents or guardians (AOR: 3.3, 95% CI:1.399–7.675, P = 0.006), Household income over 150,000 Uganda Shillings monthly (AOR: 0.3, 95% CI: 0.126–0.937, p = 0.037), 5–10 past year admissions (AOR:3.1, 95% CI: 1.651–5.749, p < 0.001). For suicidality,being aged 13–17 years (AOR:2.3, 95% CI:1.398–3.809, p = 0.001), Medication side effects (AOR:1.6, 95% CI:1.105–6.121; p = 0.039).

**Conclusion:**

1 in 3 children aged 9–17 years were depressed. Older children and adolescents living with SCA had a higher risk of depression and suicidality. Findings highlight the magnitude of mental health challenges,and underline the need for integrated mental health screening within chronic disease management.

## INTRODUCTION

Although depression is a frequently under-recognized medical condition, it has widespread impact, it accounts for 2.5% adjusted life years globally[1]. The global point prevalence rate of depression in adolescents was found to be 34% from 2001 to 2020 by Shorey et al[2]. WHO projected the condition to be the first ranking disease burden by 2030. Today, one in three Ugandans suffer from depression, which is similar to the high prevalence among undergraduate medical students in Nigeria[3, 4].

Depression therefore has either an independent or interrelation with chronic diseases such as sickle cell disease as noted by Asalman et al, who found its prevalence at 50% among patients with SCD [5]. As far as the factors associated with depression are concerned, Alsubaie et al concluded that socio-demographic factors were not significant predictors of depression in the patients with SCD [6]. The study conducted by Ezenwosu et al also concluded that depression in children and adolescents with SCA increased with increasing age and educational level[7]. Additionally, Poulose et al, showed that Jamaican adolescents with SCD had significantly higher rates of negative body satisfaction and depressive symptoms plus a nearly twice the rate of attempted suicide compared to their healthy peers [8]. This shows a relationship between depressive symptoms from low appreciation of personal physical image plus frequent pain admission pose a significant risk for suicidality

In Sudan, Salih’s study among school age children with sickle cell anemia noted that sickle cell disease had many social and psychological problems which included; enuresis, depressive symptoms, school absenteeism, and deterioration in school performance [9]. Additionally, Lukoo et al in a Congo Kinshasa based research study, reported that symptoms of depression were observed in 86.4% and 23.5% had suicidal ideation among children with sickle cell disease aged 8 to 17 years. [10]. This prevalence is a significant portion that requires exploration in our study population which shares geographical similarities with the Congo.

In Uganda, the closest publication on depression was carried out in adult patients with sickle cell disease by Mubangizi et al in Mulago Hospital. Their cross-sectional study identified prevalence of depression in that population was high with up to two thirds of patients having some form of depression. The major risk factors observed in that study include low level of education, low social support, pain crises in the past month and hospital admissions in the last 6 months [11]. Research on depression and suicidality among children and adolescents with sickle cell disease (SCD) in Uganda is limited. While depression is prevalent among individuals with chronic illnesses, specific data on children and adolescents with SCD in Uganda is lacking. Most studies use self-administered evaluation tools, and although some have employed the MINI Kid tool, these studies were conducted in different geographical locations, not reflective of Uganda’s context. Self-reported depressive symptoms scales have limitations in detecting major depression based on the Diagnostic and Statistical Manual of Mental Disorders (DSM) criteria. In Low resource populations, these studies may not provide an accurate estimate of major depression prevalence in SCD [12]. This study aimed to assess depression and suicidality in children and adolescents diagnosed with sickle cell disease (SCD). It was seeking to fill an existing knowledge gap by screening patients aged 9 to 17 years who were attending follow-up clinics at Mbale Regional Referral Hospital in Eastern Uganda. The objective was to determine the prevalence of depression and suicidality in this population, as well as to identify associated factors.

This study was seeking to identify patients who were requiring counseling and treatment for major depressive disorder and suicidality. It was enhancing clinicians’ understanding of the necessity of screening for depression in children and adolescents with sickle cell disease (SCD) and was facilitating their access to appropriate care. Additionally, the findings were informing health policy for SCD patients and were promoting future research on related comorbidities. The study included participants aged 9 to 17 with sickle cell disease at the Sickle Cell Clinic in Mbale Regional Referral Hospital, Eastern Uganda.

## METHODOLOGY

### Study Design

This study was a cross-sectional design to assess the prevalence and associated factors of depression and suicidality among children and adolescents with sickle cell anaemia (SCA) in Eastern Uganda. This design was chosen because it allows for a snapshot evaluation of the population at a single point in time, which is suitable for estimating prevalence.

#### Study Area

The study was conducted at Mbale Regional Referral Hospital in Eastern Uganda, which serves as a referral centre for several districts in the eastern region of Uganda. We chose this site because it caters to more than 1,380 patients with sickle cell anaemia (SCA), mainly children and youths, and it oversees the region’s satellite clinics, making it ideal for our research. Patients at the clinic receive services such as clinical reviews and medications, including hydroxyurea, folic acid, Fansider, or chloroquine for malaria prophylaxis and penicillin V for children under five years of age. All the services provided at the clinic are free of charge.

#### Study population

We targeted all children and adolescents with sickle cell anaemia with confirmed HbSS DNA PCR diagnosis, aged between 9 and 17 years, and attending the sickle cell clinic at Mbale Regional Referral Hospital. We also included those admitted to the ward with the same diagnosis but had improved clinically.

#### Eligibility criteria

The study included children and adolescents aged 9 to 17 years who had been diagnosed with sickle cell anaemia using PCR at the Central Public Health Laboratory in Butabika, Kampala. Participants were recruited from the Sickle Cell Clinic at Mbale Regional Referral Hospital. This age range was chosen to encompass both childhood and adolescence, as defined by the MINI KID tool.

To be eligible for the study, participants needed to be clinically stable; Participants were expected not to be in a painful crisis or in need of emergency interventions, such as blood transfusions. They were also required to be able to speak and understand the local language into which the study materials had been translated (Lumasaba, Luganda, or English). And provide assent with parental or guardian consent. Exclusion criteria included being in critical condition requiring intensive care, experiencing severe pain during data collection, or having cognitive impairment that would prevent participation.

#### Sampling Technique and Sample size

The sample size was estimated by using the OpenEpi calculator[13]. We assumed a 50% proportion of depression and suicidality because there was no other existing study specific to our study population and a 95% confidence interval, resulting in 384 participants. An additional 38 participants (10%) were included to accommodate potential non-responses. Participants were consecutively sampled based on eligibility criteria until the entire sample was achieved. The sampling process involved pre-screening all arrivals at the clinic for eligibility and the ability to consent and assent. Then, the research assistants enrolled eligible participants in order of arrival to prevent any perception of favoritism or bias. They assigned identification numbers to enrolled participants to prevent repeat enrolment by another research assistant during subsequent follow-up visits. This routine was maintained until the daily enrolment target was met.

### Study Variables

Our dependent variables were depression and suicidality, which were measured via the MINI KID [14]. The independent variables were age, crisis episode duration, sex, household income, education level, frequency of admissions, and routine care.

#### Recruitment and data collection procedure

Following the successful sampling of participants in the sickle cell clinic held every Wednesday, which started on 01/02/2024 and ended on 05/06/2024. The research assistant sought and completed a written consent and assent form from the participants and parents or guardians, respectively. They also obtained a signature or thumbprint from the participants during enrolment. The data collection tool was in three parts, namely: the demographic data section, the depression screening, and the suicidality section from the M.I.N.I. KID Tool. It was an interviewer-administered tool in hard copy, where the interviewer marked and recorded responses from enrolled participants.

Participants within the eligible age range were first identified, and the study was explained during clinic health education sessions. Consent from parents or guardians and assent from children were obtained. Non-participants continued receiving routine care. Consenting participants were interviewed in a private setting for 30–60 minutes, with confidentiality assured.

Trained research assistants (clinical officers and medical students) conducted interviews using the MINI-KID tool after receiving training from a psychiatrist. Data were then transferred to the electronic version into the Kobo tool for analysis.

#### Data collection tools

We created a demographic section to capture participants’ clinical data, borrowed from validated tools such as demographic health survey reports. For depression and suicidality, we utilized the M.I.N.I. International Neuropsychiatric Interview for Children and Adolescents (M.I.N.I KID), English version 7.0,2 [14], aligned with DSM-5 criteria. Originally designed for DSM-IV disorders, the MINI-KID has been updated for the DSM-TR and organized into diagnostic sections via a branching-tree logic that includes two screening questions for each disorder.

We were interested in sections A and B, which assess depression and suicidality. To diagnose major depressive episodes, question A4 “Did these sad, depressed feelings cause many problems at home, at school, or with friends?” must be answered “YES,” along with five additional positive responses for a past or current episode. If A5 is also yes, a recurrent episode diagnosis is made. Scores for suicidality are categorized as 1–8 points for low risk, 9–16 points for moderate risk, and above 17 points for high risk. The MINI-KID is a valuable tool with several noteworthy psychometric properties. It shows substantial to excellent concordance with the K-SADS-PL across various diagnoses, including mood disorders, anxiety disorders, substance use disorders, ADHD or behavioural disorders, and eating disorders. The tool demonstrated significant sensitivity for 15 out of 20 DSM-IV disorders and excellent specificity for 18 disorders. Overall, the MINI-KID provides reliable and valid psychiatric diagnoses for children and adolescents[15]. The same tool was used to validate the CRAFFT study, a tool in Kampala and Mbale, Uganda, that focuses on substance use disorders[16]. It has also been used in child mental health STC studies in Uganda and Burkina Faso[17]. The sociodemographic and clinical factors questionnaire was developed specifically for this study, guided by the national social demographic survey tools.

### Data management and analysis

The data was downloaded from kobo software in Microsoft Excel software and checked for errors before being exported to Stata version 17 for analysis (http://www.stata.com/stata17/). Continuous descriptive variables are presented as the means and standard deviations. Categorical variables are presented as proportions. We performed bivariable and multivariable logistic regression to determine the associations between the explanatory variables and the outcome variables, depression and suicidality. Selection of variables for multivariable analysis was guided primarily by prior literature, clinical relevance, and biological plausibility, together with evidence from bivariable analysis at p ≤ 0.20 (as long as they were not in the causal pathway and were not strongly collinear with other independent variables) (Hosmer Jr, 2013; Hosmer Jr *et al*., 2013). Collinearity was assessed using the variance inflation factor, and variables with a variance inflation factor greater than 10 were considered strongly collinear. Where collinearity was identified, the variable with greater conceptual and clinical relevance to the outcome was retained in the multivariable model.

A backward elimination approach was then applied cautiously to obtain a more parsimonious model from the set of variables identified a priori and from bivariable analysis which included the age, level of education, house hold income,employment status of the parents or guardiansand medication side efects, while also assessing possible confounding. Variables removed from the model were re evaluated for confounding and were retained if their exclusion changed the measure of association by 10% or more. Given the relatively limited number of outcome events, particularly for suicidality, care was taken to avoid overfitting by restricting multivariable models to variables considered most relevant based on prior evidence and observed associations. The final model included variables retained on the basis of statistical contribution and confounding assessment, and model fit was assessed using the Hosmer–Lemeshow goodness of fit test (Hosmer, 1989; Simonetti *et al*., 2017).

### Ethical Consideration

The study was approved by the Busitema University Research Ethics Committee (BUFHS-2023-140) and the Mbale Regional Referral Hospital Ethics Committee (MRRH-2023–140). Informed consent was obtained from parents or guardians, and assent was obtained from the participants, as all participants were under 18 (minors per Ugandan law). Trained research assistants collected data to prevent conflicts of interest. Confidentiality was maintained by securely storing participant information and assigning unique identification numbers. The electronic patient data were password protected. A psychiatrist was involved in supervising the study and ensured that participants diagnosed with depression or suicidality received appropriate evaluation and care from the mental health professionals at Mbale Regional Referral Hospital, including counseling and pharmacologic treatment. The study adhered to the principles of the Declaration of Helsinki, prioritizing the rights and well-being of participants.

## Results

A total of four hundred and twenty-two participants were enrolled in the study from a population of one thousand eight hundred and seventy-five people attending the MRRH Sickle Cell Clinic. Out of these, 421 participants were successfully included in the study, while one thousand four hundred and fifty-four clinic attendees were excluded.

### Sociodemographic characteristics of the participants

The children in the study ranged from a minimum of 9 years to a maximum of 17 years, with a mean age of 12.5 years and a standard deviation of 2.8 years. As shown in the table below, female respondents (56.3%) outnumbered male participants (43.7%). Most participants (74.3%) were living with both parents, the majority of the parents were unemployed (76.2%), and 43.7% were at the upper primary education level.

A higher proportion of adolescents aged 13–17 years were depressed, accounting for 55.2% (74/134) of those with depression compared to 41.5% (119/287) of those without depression. In contrast, children aged 9–12 years had a lower prevalence of depression at 44.8% (60/134), while they made up a larger proportion of those without depression at 58.5% (168/287).

The highest level of education also showed a significant association with depression (p = 0.001). Participants with no education had the lowest rates of depression, with none reporting depressive symptoms. Those in lower primary education had a significantly lower prevalence of depression at 32.6% (42/134) compared to 50.9% (141/287) among those without depression. In contrast, those in secondary education showed a higher prevalence of depression at 20.2% (26/134) compared to 7.2% (20/287) among those without depression.

Education level was also significantly associated with suicidality (p = 0.001). Participants in lower primary education had a lower prevalence of suicidality at 26.0% (20/80) compared to 49.5% (163/341) among those without suicidality. Details in Table 1

### Prevalence of major depression and suicide

One-third (31.8%, n = 134) (95% CI 27.4% − 36.5%) of the study participants reported experiencing major depression. The most frequently observed pattern was “past, recurrent” episodes, noted by 40.3% (n = 54) of the participants. Additionally, 21.6% (n = 29) had experienced depression in the past but were not currently depressed. Among the participants who had major depressive episodes, 35.8% (n = 48) were depressed at the time of the study

With respect to suicidality, 19.0% (n = 80; 95% CI 15.4% − 23.1%) reported experiencing suicidal thoughts, 21.3% (n = 17) of the 80 participants stated that they had attempted suicide at least once, and 3.7% indicated that they might have suicidal thoughts in the near future. Furthermore, 21.2% (n = 17) of the sample points were categorized as very high severity for suicidality (> 17 points), whereas 55.0% (n = 44) scored lower points, 62.5% (n = 50) of the 80 participants who were suicidal had depression, with a P value < 0.001, as shown in the Table 2.

### Factors associated with depression among children with sickle cell anemia

Children and adolescents who attained secondary education had 3.5 times the odds of depression as those with no formal education [adjusted odds ratio (AOR) of 3.5 (95% confidence interval: 1.965–12.756), p-value = 0.049].

Children and adolescents whose parents or guardians were formally employed had 3.3 times the odds of experiencing depression as those whose parents or guardians were not formally employed. [Adjusted odds ratio (AOR) 3.3, 95% CI: 1.399–7.675, P value = 0.006]. Additionally, the children and adolescents from households with a monthly income of more than 150,000 Uganda Shillings (approximately 40.65 USD) were 70% less likely to experience depression compared to those from households with a monthly income of less than 10,000 Uganda Shillings (approximately 2.7 USD), with an [AOR 0.3, 95% CI: 0.126–0.937, P value = 0.037].

Children and adolescents with 5 to 10 admissions had 3.1 times the odds of depression as those who did not have any admissions in the past twelve months. [AOR 3.1, 95% CI: 1.651–5.749, P value < 0.001], as shown in the Table 3.

### Factors associated with suicidality among children with sickle cell anemia

Children and adolescents aged 13 to 17 years had 2.3 times the odds of experiencing suicidality as those aged 9 to 12 years, with an AOR of 2.3 (95% CI: 1.398–3.809) and a P value of 0.001. Additionally, children and adolescents who experienced side effects from medications were 2.6 times the odds of suicidality as those who did not experience any side effects. The AOR for this group was 1.6 (95% CI: 1.105–6.121), with a P value of 0.039. Table 4

## Discussion

This study demonstrates a substantial mental health burden among children and adolescents with sickle cell anaemia (SCA) in Eastern Uganda, with notably high prevalences of depression (31.8%) and suicidality (19.0%). These findings underscore the profound psychological impact of living with a chronic, painful, and unpredictable condition[18]. Recurrent vaso-occlusive crises, frequent hospitalizations, and the lifelong nature of SCA likely contribute to persistent emotional distress, reinforcing prior evidence that chronic illness in childhood is strongly associated with mood disorders[19].

The prevalence of depression observed in this study is higher than reports from similar settings, such as studies in Nigeria[20, 21], but lower than findings from the Democratic Republic of Congo[10]. These discrepancies may reflect methodological differences, including the use of more sensitive diagnostic tools such as the MINI-KID in our study, as well as contextual variations in healthcare access, socioeconomic conditions, and psychosocial support systems. Environmental and economic constraints, may further exacerbate depressive symptoms in this population.

The prevalence of suicidality(19%), is particularly concerning, especially given that a large proportion of affected participants 21.3% had attempted suicide within the past year. This suggests that depressive symptoms may remain unrecognized and untreated, progressing to more severe outcomes[22]. Older age was significantly associated with suicidality, likely reflecting increased disease awareness, longer duration of illness, and the added psychosocial stressors of adolescence, including hormonal changes in adolescence and self image formation. Similarly noted by Hassett et al in 2014, Carballo et al in 2020 and Bhatt-Poulose et al in 2016 respect**i**vel**y** [8, 23–25]

Our findings also highlight the complex interplay between treatment-related factors and mental health. Medication side effects were significantly associated with suicidality, suggesting that treatment burden and adverse experiences may negatively influence adherence and psychological well-being as noted by Walsh K. E *et al* in 2014[26]. Although no specific medication was identified, the cumulative effect of multiple therapies may contribute to distress and fear surrounding treatment like it was seen in a study by Stübner *et al*.among adolescents who were taking antidepressant medications[27]. Notably, the children and adolescents attempted suicide using routine medications provided at the clinic, similar to that seen in the studies by Mishkin AD et al and Swirsky et al [7, 28].

Socioeconomic and family-related factors were also important determinants. Higher levels of education were associated with increased odds of depression, possibly due to greater awareness of the chronic and incurable nature of SCA. Conversely, higher household income appeared protective, likely by improving access to healthcare, pain management, and supportive resources. Interestingly, children of formally employed caregivers had higher odds of depression, which may reflect reduced caregiver availability and emotional support despite improved financial stability.

This finding is similar to those from studies by Marsh et al., Adzika VA et al ,and Ekoube *et al*., [18, 29, 30]. This is also noted in both studies in America and Nigeria on the economic burden of sickle cell disease[31, 32].

Frequent hospital admissions were another significant predictor of depression, emphasizing the cumulative psychological toll of repeated illness episodes and healthcare encounters. These disruptions may impair academic performance, social integration, and overall quality of life, further contributing to depressive symptoms.

Overall, these findings highlight the urgent need for integrated mental health services within SCA care programs. Routine screening for depression and suicidality, alongside psychosocial support for both patients and caregivers, is essential. Addressing socioeconomic disparities and treatment-related challenges may further mitigate the mental health burden in this vulnerable population.

### Strengths and Limitations of the Study

Our study was sufficiently powered to examine the prevalence of depression and suicidality, as well as the various demographic, clinical, and economic factors associated with these issues. We also used a validated tool for the study process. However, we encountered a few limitations in our research, namely, the study population consisted only of patients who were actively seeking care (clinic-based sample), which excluded individuals who were not attending the clinic, which could affect the study results at the community level screening for those sickle cell anaemia patients who do not attend clinics. Additionally, there was the potential for recall bias among patients, although this was minimized by focusing on symptoms experienced over the past 12 months, which were readily available in their clinical records.

## Conclusion and Recommendations

Older children and adolescents with sickle cell anaemia are at increased risk of depression and suicidality, influenced by factors such as higher education, parental employment, low household income, frequent hospital admissions, and medication side effects. These findings highlight the need for routine mental health screening and timely interventions in this population. Despite limitations of single-site sampling, our study included participants from multiple districts (17 out of 30), supporting the relevance of the findings. In a setting with limited mental health resources, the high burden observed underscores the urgent need for integrated psychosocial support for children with SCA in Eastern Uganda.

We recommend the update of Uganda’s national policy for Sickle Cell Anaemia/Disease (SCD) management in children to include: Routine screening for depression as soon as patients reach a higher level of education, and to offer psychological support as well as emphasize close parental or guardian involvement in the follow up and reporting clinical outcomes of their children. Further research on the prevalence of depression and suicidality in other chronic diseases is highly recommended.

## Figures and Tables

**Table 1:** Baseline sociodemographic and clinical characteristics of the participants stratified by depression and suicidality. (n = 421)

Variable	Total N = 421 (%)	Depression		P-value	Suicidality		P-value
		No n = 287 (%)	Yes n = 134 (%)		No n = 341 (%)	Yes n = 80 (%)	
Gender				0.345			0.257
Female	237 (56.3)	166 (57.8)	71 (53.0)		187 (54.8)	50 (62.5)	
Male	184 (43.7)	121 (42.2)	63 (47.0)		154 (45.2)	30 (37.5)	
Age				0.008			0.001
9–12	228 (54.2)	168 (58.5)	60 (44.8)		198 (58.1)	30 (37.5)	
13–17	193 (45.8)	119 (41.5)	74 (55.2)		143 (41.9)	50 (62.5)	
Highest level of education				0.001			0.001
No education	18 (4.3)	13 (4.5)	5 (3.7)		14 (4.1)	4 (5.0)	
Lower Primary	183 (43.5)	141 (49.1)	42 (31.3)		163 (47.8)	20 (25.0)	
Upper Primary	173 (41.1)	113 (39.4)	60 (44.8)		131 (38.4)	42 (52.5)	
Secondary	47 (11.2)	20 (7.0)	27 (20.1)		33 (9.7)	14 (17.5)	
Staying with				0.675			0.668
Both parents	313 (74.3)	214 (74.6)	99 (73.9)		255 (74.8)	58 (72.5)	
Guardian	37 (8.8)	23 (8.0)	14 (10.4)		31 (9.1)	6 (7.5)	
Single parents	71 (16.9)	50 (17.4)	21 (15.7)		55 (16.1)	16 (20.0)	
Employment status of parent/Guardian				0.119			0.128
No	320 (76.2)	225 (78.4)	95 (71.4)		265 (77.7)	55 (69.6)	
Yes	100 (23.8)	62 (21.6)	38 (28.6)		76 (22.3)	24 (30.4)	
Major income-generating activity for your family				0.849			0.446
Parents Formally employment	70 (16.7)	49 (17.1)	21 (15.8)		53 (15.6)	17 (21.2)	
Parents doing business	82 (19.6)	54 (18.9)	28 (21.1)		66 (19.5)	16 (20.0)	
Peasant	267 (63.7)	183 (64.0)	84 (63.2)		220 (64.9)	47 (58.8)	
Estimated household monthly income				0.12			0.661
< 10,000	31 (7.4)	16 (5.6)	15 (11.2)		26 (7.6)	5 (6.2)	
10,000 to 50,000	158 (37.5)	112 (39.0)	46 (34.3)		132 (38.7)	26 (32.5)	
50,001 to 150,000	101 (24.0)	69 (24.0)	32 (23.9)		81 (23.8)	20 (25.0)	
> 150,001	131 (31.1)	90 (31.4)	41 (30.6)		102 (29.9)	29 (36.2)	
Number of admissions in the past 12 months				0.010			0.496
None	52 (12.4)	37 (12.9)	15 (11.2)		45 (13.2)	7 (8.8)	
< 5	302 (71.7)	217 (75.6)	85 (63.4)		245 (71.8)	57 (71.2)	
5 to 10	58 (13.8)	28 (9.8)	30 (22.4)		44 (12.9)	14 (17.5)	
10 to 15	7 (1.7)	4 (1.4)	3 (2.2)		6 (1.8)	1 (1.2)	
> 15	2 (0.5)	1 (0.3)	1 (0.7)		1 (0.3)	1 (1.2)	
Experienced side effects				0.159			0.033
No	399 (94.8)	275 (95.8)	124 (92.5)		327 (95.9)	72 (90.0)	
Yes	22 (5.2)	12 (4.2)	10 (7.5)		14 (4.1)	8 (10.0)	

Objective: To determine the prevalence of depression and suicidality among children and adolescents with SCD attending the clinic at Mbale Regional Referral Hospital.

**Table 2:** Prevalence of Depression and Suicidality among Children with Sickle Cell Anemia at Mbale Regional Referral Hospital (n = 421)

Major depression	Frequency	Percentage
	
No	287	68.2
Yes	134	31.8
Major depression episode		
Past, current, and recurrent	43	32.1
Current	1	0.8
Past and current	4	3.0
Past	29	21.6
Past and recurrent	54	40.3
Recurrent	3	2.2
Suicidality		
No	341	81.0
Yes	80	19.0
Type of Suicidality		
Current	60	75.0
Lifetime attempt	17	21.3
Likely in the near future	3	3.7
Suicidality Score		
1–8points	44	55.0
9–16points	19	23.8
> 17points	17	21.2
Suicidal behaviour disorder		
No	413	98.1
Yes	8	1.9
Suicidal behaviour disorder types		
Current	4	50.0
In early remission	1	12.5
In remission	2	25.0
Lifetime attempt	1	12.5

Specific objective: To identify the factors associated with depression and suicidality in the study population.

**Table 3:** Factors associated with major depressive episodes among children with sickle cell anemia (n = 421)

Variable	COR (95% CI)	P value	AOR (95% CI)	P value
**Age**				
07–12	1		1	
13–17	1.7 (1.151–2.633)	0.009	1.1(0.59–1.872)	0.866
**Education level**				
No formal education	1		1	
Lower primary	0.8 (0.261–2.298)	0.645	0.6 (0.197–1.773)	0.348
Upper primary	1.4 (0.47–4.057)	0.558	1.1(0.365–3.24)	0.881
Secondary	3.5 (1.076–11.451)	0.037	3.5(1.965–12.756)	**0.049**
**Parent/guardian formally employed**				
No	1		1	
Yes	1.5 (0.908–2.322)	0.120	3.3 (1.399–7.675)	**0.006**
**Major income-generating activity for family**				
Formally employed	1		1	
Parents doing business	1.2 (0.61–2.401)	0.586	2.7 (0.99–7.486)	0.052
Peasant	1.1 (0.604–1.899)	0.814	2.6 (0.978–7.051)	0.055
**Estimated household monthly income**				
< 10,000	1		1	
10,000 to 50,000	0.4 (0.2–0.959)	0.039	0.5 (0.205–1.206)	0.122
50,000 to 150,000	0.5 (0.218–1.123)	0.092	0.5 (0.18–1.222)	0.121
> 150,000	0.5 (0.219–1.076)	0.075	0.3 (0.126–0.937)	**0.037**
**Number of admissions in the past 12 months**				
None	1		1	
< 5	1.0 (0.504–1.851)	0.917	0.7 (0.349–1.353)	0.278
5 to 10	2.6 (1.199–5.827)	0.016	3.1 (1.651–5.749)	**< 0.001**
10 to 15	1.9 (0.369–9.28)	0.455	2.6 (0.562–11.98)	0.222
> 15	2.5 (0.145–42.05)	0.533	2.3 (0.126–43.141)	0.57

**COR** -*Crude Odds Ratio*, **AOR**-*Adjusted Odds Ratios*, **Formally employed**-*Salaried eaners*, **Parents doing business**-*Any payment for goods or services rendered by an individual*, **Peasant**-*a person who mainly depends on small scale farming for livelihood*

**Table 4:** Factors associated with suicidality among children with sickle cell anemia (n = 80)

Variable	COR (95% CI)	P value	AOR (95%: CI)	P value
**Age**				
9–12	1		1	
13–17	3.52 (1.799–6.876)	<0.001	2.3(1.398–3.809)	**0.001**
**Education level**				
No formal education	1		1	
Lower Primary	0.46 (0.133–1.56)	0.21	0.4(0.129–1.432)	0.169
Upper Primary	1.92 (1.284–2.95)	0.02	1.1(0.350–3.595)	0.846
Secondary	1.05 (1.282–3.93)	0.008	1.5(0.415–5.314)	0.543
**Parent employed**				
No	1		1	
Yes	1.31 (1.517–3.317)	0.04	1.5(0.884–2.619)	0.13
**Major income-generating activity**				
Formally employed	1		1	
Parents doing business	1.85 (0.294–3.468)	0.768	0.8(0.349–1.636)	0.477
Peasant	1.92 (0.316–4.689)	0.002	1.7 (0.355–3.251)	0.207
**Experienced side effects**				
No	1		1	
Yes	2.79 (1.617–5.164)	0.005	1.6(1.105–6.121)	**0.039**

**COR** -*Crude Odds Ratio*, **AOR**-*Adjusted Odds Ratios*, **Formally employed**-*Salaried eaners*, **Parents doing business**-*Any payment for goods or services rendered by an individual*, **Peasant**-*a person who mainly depends on small scale farming for livelihood*

## Data Availability

The datasets generated during and/or analysed during the current study are available from the corresponding author on reasonable request.
